# –A cross-sectional study of clinical learning environments across four undergraduate programs using the undergraduate clinical education environment measure

**DOI:** 10.1186/s12909-021-02687-8

**Published:** 2021-05-05

**Authors:** Malin Sellberg, Per J. Palmgren, Riitta Möller

**Affiliations:** 1 Department of Clinical Science, Intervention and Technology, Karolinska Institutet, Stockholm, Sweden; 2 Functional Area Occupational Therapy and Physiotherapy, Allied Health Professionals Function, Karolinska University Hospital, Huddinge, 171 76 Stockholm, Sweden; 3 Department of Learning Informatics, Management and Ethics, Karolinska Institutet, 171 77 Stockholm, Sweden; 4 Department of Medical Epidemiology and Biostatistics, Karolinska Institutet, 171 77 Stockholm, Sweden

**Keywords:** Clinical learning environment, Evaluation, Supervision, Undergraduate, Medical, Nursing, Physiotherapy, Speech-language pathology

## Abstract

**Background:**

The clinical learning environment (CLE) influences students’ achievement of learning outcomes and the development of their professional behaviors. However, CLEs are not always optimal for learning because of clinical productivity expectations and a lack of support from supervisors. The purpose of this study was to describe and compare students’ perceptions of their CLEs across four undergraduate programs.

**Methods:**

This study is cross-sectional. In total, 735 students who were registered in the medical, nursing, physiotherapy, and speech-language pathology (SLP) programs were invited to participate. Data were collected using an online survey, which included demographics and the Undergraduate Clinical Education Environment Measure (UCEEM). The UCEEM consists of 26 items congregated into two overarching dimensions—experiential learning and social participation—with four subscales: opportunities to learn in and through work and quality of supervision, preparedness for student entry, workplace interaction patterns and student inclusion, and equal treatment.

**Results:**

In total 280 students (median age 28; range: 20–52; 72% females) returned the questionnaire. The mean total UCEEM score was 98.3 (SD 18.4; range: 91–130), with physiotherapy students giving the highest scores and medical students the lowest. The mean scores for the dimensions experiential learning and social participation for all the students were 62.8 (SD 13.6; range 59–85) and 35.5 (SD 6.2; range 13–45), respectively. Medical students rated the lowest for all subscales. The items receiving the highest ratings concerned equal treatment, whereas those receiving the lowest ratings concerned supervisors’ familiarity with the learning objectives. There were few statistically significant differences between the semesters within each program.

**Conclusions:**

The students generally hold positive perceptions toward their CLEs. However, the students from the medical and nursing programs rated their learning environment lower than did the students from the physiotherapy and SLP programs. Importantly, in several aspects, the medical students provided significantly lower ratings for their CLE compared with the students from the other programs. The medical students’ low ratings for their supervisors’ familiarity with the learning objectives underscore the need to ensure that the prerequisites for optimal supervision are met.

## Background

The learning environment (LE) of healthcare professionals is primarily shaped by the interactions between different stakeholder groups and the organizational structures of the environment. While the existing literature describes the importance of the LE, it often lacks a comprehensive explanation of what constitutes this environment [1]. Aspects such as social relationships, institutional culture, physical space, infrastructure, supervision, and formal and informal curricula all belong to the LE [2]. The clinical learning environment (CLE), in turn, refers to the clinical workplace in which health professions students complete their clinical placements as part of their education [3]. The CLE is considered important, as it is in this context that health professions students apply theoretical knowledge to practice, acquire clinical skills, and develop problem-solving and clinical reasoning skills [4, 5]. However, exploring the CLE can be complex because it may encompass a multitude of settings, features, and stakeholders. Students comprise one key stakeholder group, and research has shown that the CLE profoundly influences their behaviors and contributes to their learning, performance, contentment, and success [1].

Learning in a clinical environment entails challenges in balancing ongoing healthcare delivery and students’ learning [6, 7]. It is widely acknowledged that the student–supervisor relationship is central to the learning experience and the achievement of learning outcomes [5, 8]. However, clinical productivity expectations and poor support from management may negatively affect supervisors’ time for clinical teaching [9, 10]. Other challenges include variations in supervisors’ preparedness, lack of feedback, and external factors, such as increased class sizes and patient availability (e.g., shorter stays at the hospital and patients with multiple comorbidities) [11]. A negative CLE experience has been associated with the quality and safety of patient care [12] and students’ quality of life [13]. Thus, a supportive CLE does not exist self-evidently [14]; it needs active maintenance and continuous assessment [15].

Empirical investigations of the CLE have been mostly conducted among medical, nursing, and dentistry students [16–18]. They frequently score the environment more positively than negatively [19]. A handful of studies have been carried out among physiotherapy students, and they show similar results [20–22]. Brown et al. [20] and Ousey et al. [21], who assessed physiotherapy students among other undergraduate students within health sciences, used the Dundee Ready Education Environment (DREEM) questionnaire, which is a universal inventory to obtain information about the LE in medical institutions and not specifically the CLE. Surprisingly, to date, scant empirical evidence exists regarding speech language pathology (SLP) students’ CLE despite clinical practice being an essential component of their education. Furthermore, to the best of our knowledge, few studies have surveyed and compared a comprehensive cohort of healthcare professional students and their perceptions of their CLEs.

Several instruments have been developed to measure the quality of the LE [23–25]. However, these were not constructed for the primary purpose of gauging the quality of the CLE [23], did not distinguish between undergraduate and postgraduate students [24, 26], or were developed for postgraduate education [27, 28]. In addition, only a few of the established instruments have been shown to be psychometrically stable and conceptually grounded in theoretical frameworks that define the phenomena that should be measured [26, 29]. The recently developed Undergraduate Clinical Education Environment Measure (UCEEM) was created to evaluate undergraduate medical students’ perceptions of the invitational, organizational, and pedagogical quality of the CLE in hospitals [30]. The measure has been reported to have good psychometric properties in diverse contexts [30, 31]. It is grounded in contemporary learning theories [30], so it was chosen for the current study.

In order to better support students’ learning and supervision, this study aims to describe and compare undergraduate students’ perceptions of their CLEs across four different undergraduate programs. Specifically, we wanted to shed light on i) the differences in students’ perceptions of their CLEs across different study programs and (ii) whether the perceptions between students from earlier and later semesters are comparable. Understanding these perceptions is a key starting point for optimizing the CLE and learning practices. This is particularly important because clinical training is a comprehensive part of health profession education.

## Methods

### Study design

This was a questionnaire-based cross-sectional study. It constituted part of a larger prospective research project investigating students’ perceptions of their CLEs, stress, and well-being.

### Setting of the study

The context of the study is a Swedish medical university. The medical program is 5.5 years (330 European Credit Transfer System, ECTS, credits; 11 semesters) and admits 165 students twice annually. The first 2 years cover mainly preclinical education (e.g., cell biology, anatomy, physiology). Clinical placements are included in almost all semesters, accounting for about 55% of the program, and are often short, with an average length of one to 2 weeks. Three so-called threads (professionalism, primary care, and scientific education) run throughout the program, comprising a total of 25 weeks of electives. The physiotherapy program is 3 years (180 ECTS credits; six semesters) and admits 65 students twice annually. In total, there are approximately 25 weeks of clinical courses clustered in the themes of professional development, assessment, intervention, health care, and scientific method. The length of clinical placements varies from one to 8 weeks. The SLP program is 4 years (240 ECTS credits; 8 semesters) and admits 40 students annually. It includes courses in psychology, linguistics, and medicine, as well as theoretical and practical courses in SLP, the clinical science of communication, and swallowing disorders. Five tracks (communication, research methods, medical science, SLP theory, and SLP practice) run throughout the program. Clinical education makes up about 20% of the program, comprising one- to three-week-long placements. The nursing program is 3 years (180 ECTS credits; 6 semesters) and admits 120 students twice annually. It includes mainly subject-integrated courses in nursing, medicine, public health sciences, and social and behavioral sciences. The program is organized into three themes and seven thematic threads (ethics, interprofessional learning, leadership and learning, patient safety, professional development, scientific development, and care on equal terms). Clinical courses are included in all semesters and make up about 50% of the program. Students usually spend five to 6 weeks on one placement. Students from all programs do a large part of their clinical training at the same university hospital. However, training is also carried out at other hospitals, health care centers, and private clinics, all of them belonging to the same health care region. Thus, the conditions for the clinical education of the students are quite different.

### Participants

Convenience sampling was used to source participants from all major health science programs offered at our university. To obtain a broad representation, we included students from shorter and longer study programs as well as those from earlier and later semesters, who, accordingly, had varying lengths of experience with their CLEs. Thus, medical students from semesters 6 and 10, physiotherapy students from semesters 3 and 6, SLP students from semesters 4 and 6, and nursing students from semesters 3 and 6 were invited to participate. A total of 735 students were registered for these semesters at the time of the study. They were informed orally and in writing about the study, which was followed up by an e-mail containing a survey link to the questionnaires and an attached information letter stating that participation was voluntary and that declining would not affect their education.

### Data collection

The online questionnaire developed specifically for this project consisted of a section on sociodemographic data (8 items) and the UCEEM (26 items). The students were asked to complete the questionnaire for their current/latest placement. In case of no reply, four reminders were sent between 4 and 10 weeks after the first questionnaire administration. Each participant received a book voucher as compensation.

### The undergraduate clinical education environment measure (UCEEM)

The UCEEM is a self-administered, closed-ended inventory relating to a variety of topics of direct relevance to the CLE [30]. It is developed through quantitative and qualitative data from focus groups, in-depth interviews with students and clinical supervisors, and feedback from key stakeholders. It is also designed and validated for the Swedish context and shows strong psychometric properties [30]. The original UCEEM comprises 25 items scored from 1 to 5 using a 5-point Likert response; where each of the statements were on on the following scale: 1 = fully disagree, 2 = agree to a slight extent, 3 = neutral, 4 = agree to a large extent, and 5 = fully agree. The revised version comprising 26 items (score range: 26–30) (personal correspondence with author 09/18/2019) was used in this study. The items are congregated into two overarching dimensions—experiential learning (A) and social participation (B)—with four subscales: A1) opportunities to learn in and through work and quality of supervision (11 items; score range: 11–55), A2) preparedness for student entry (6 items; score range: 6–30), B1) workplace interaction patterns and student inclusion (6 items; score range: 6–30), and B2) equal treatment (3 items; score range: 3–15). For all item and subscale scores, a higher score indicates a more positive response.

### Data analysis

The UCEEM items were analyzed at the individual, subscale, and overall levels and reported as averages through means. The item response rate (IRR) was calculated as the proportion of respondents who completed all items of the questionnaire. The IRR at the item level was > 90%, which was considered satisfactory [32]. The Mann-Whitney U test was conducted to compare the results for gender, age, previous university studies, students with or without children, and between semesters within each program. The Kruskal–Wallis tests with Dunn–Šidák post-hoc tests were performed to compare different study programs. The level of significance was set to 0.05. *P*-values were adjusted for multiple comparisons by using Bonferroni adjustments of primary endpoints to control for the risk of mass significance. Cronbach’s alpha was used to assess the internal consistency of the subscale scores of the UCEEM, and a minimum alpha coefficient of 0.70 was used to indicate an adequate level of consistency [33]. In line with the suggestion by Swift et al. [34] on how to cluster aggregated Likert responses, we considered items with a mean score > 4.5 as representing particular strong areas, those with a mean score ≤ 3 as needing particular attention, and those with a mean score between 3 and 4 as indicating LE areas that could be improved. However, the 1scoring limits were adjusted to a Likert response of 1–5; comparatively, Swift et al. [34] used a scale of 1–4. The statistical analyses were performed using SPSS version 26 or R version 3.4.1.

## Results

### Participants

**Fig. 1:**
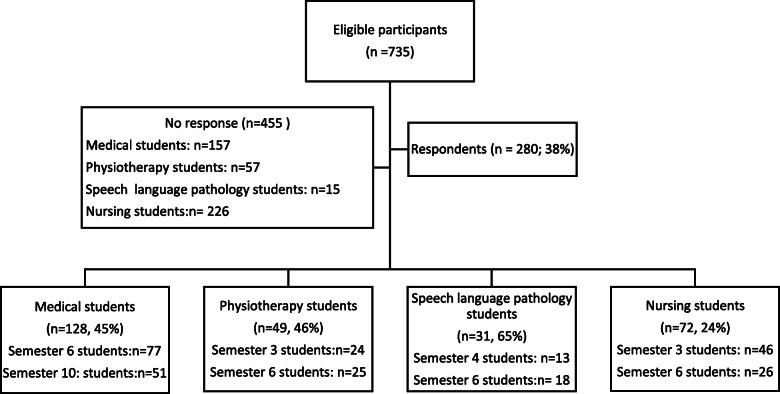
Flow chart showing the number of participants and the response rates (n, %) in the study

A total of 280 students (median age 26 years; 72% females) returned the questionnaire, which corresponded to a response rate of 38% (Fig. 1, Table 1). There were no differences between the participants and the nonparticipating students from the same semesters in terms of gender and age (data not shown).

**Table 1:** Sociodemographic characteristics of all the participants (*n = 280*)

	All students ***n*** (%)	Medical students ***n*** (%)	Physiotherapy students ***n*** (%)	Speech-language pathology students ***n*** (%)	Nursing students ***n*** (%)
**Number of students**	280 (100)	128 (46)	49 (17)	31 (11)	72 (26)
From earlier semesters	160 (57)	77 (60)	24 (49)	13 (42)	46 (64)
From later semesters	120 (43)	51 (40)	25 (51)	18 (58)	26 (36)
**Number of females**	202 (72)	80 (62)	35 (71)	28 (90)	59 (82)
**Age,** years (median IQR)	26 (23–32)	26 (24–30)	25 (23–29)	28 (24–33)	29 (24–36)
**Students with children**	59 (21)	18 (14)	7 (14)	8 (26)	26 (36)
**Previous working experience**	191 (68)	76 (59)	27 (55)	25 (81)	56 (78)
**Previous university studies**	160 (57)	76 (59)	27 (55)	16 (52)	41 (57)

*Abbreviations*:*n* The number of participants, *IQR* Interquartile range

### Total UCEEM scores

A summary of the data on the total UCEEM, overarching dimension, and subscale scores is presented in Table 2. The mean total score for all students was 98.3 (SD: 18.4; range: 91–130), with physiotherapy students giving the highest scores and medical students giving significantly lower scores than all the other students. The scores did not differ significantly between female (98.4) and male (98.3) students, younger (98.9) and older students (98.2), or students with or without children (100.0 and 98.2, respectively) (data not shown). However, the mean ratings were significantly lower (*p* <  0.01) among students with previous university studies (96.1) than those without (101.2).

**Table 2:** The scores (mean, SD) for the UCEEM total, the scales, and the items for all students (*n = 280*)

UCEEM overarching dimensions, subscales, and items	All students Mean (SD) *n = 280*	Medical students Mean (SD) *n = 128*	Physiotherapy students Mean (SD) *n = 49*	Speech-language pathology students Mean (SD) *n = 31*	Nursing students Mean (SD) *n = 72*	M-PT *p* value	M-SLP *p* value	M-N *p* value	PT-SLP *p* value	PT-N *p* value	SLP-N *p* value
**Total score**^a^	98.3 (18.4)	90.7 (14.8)	112.3 (12.9)	108.4 (17.7)	97.9 (20.3)	< 0.001	< 0.001	< 0.01	ns	< 0.001	< 0.01
**Overarching dimension Experiential learning** (A1 + A2)	62.8 (13.6)	57.0 (10.8)	73.5 (9.8)	70.1 (13.0)	62.7 (14.6)	< 0.001	< 0.001	< 0.01	ns	< 0.001	< 0.05
**Subscale A1: Opportunities to learn in and through work and quality of supervision**	41.1 (8.6)	37.8 (7.2)	47.0 (7.0)	44.9 (8.6)	41.3 (9.2)	< 0.001	< 0.001	< 0.01	ns	< 0.001	ns
3. My (work) tasks are relevant to the learning objectives.	3.9 (0.9)	3.6 (1.0)	4.4 (0.7)	4.2 (0.8)	4.0 (1.1)	< 0.001	< 0.01	< 0.01	ns	< 0.05	ns
4. I am sufficiently occupied with meaningful (work) tasks.	3.6 (1.1)	3.2 (0.9)	4.1 (1.0)	4.1 (1.0)	3.5 (1.1)	< 0.001	< 0.001	< 0.05	ns	< 0.01	< 0.05
5. My tasks are suitably challenging for my level of knowledge and skills.	3.7 (1.0)	3.5 (1.0)	4.0 (1.0)	4.2 (0.9)	3.7 (1.1)	< 0.001	< 0.001	ns	ns	< 0.05	< 0.05
6. I am encouraged to participate actively in the work here.	3.9 (1.0)	3.4 (0.8)	4.6 (0.8)	4.4 (0.7)	3.9 (1.0)	< 0.001	< 0.001	< 0.001	ns	< 0.001	< 0.05
13. I receive useful feedback from my supervisors.	3.6 (1.2)	3.1 (1.1)	4.5 (0.7)	4.0 (1.2)	3.8 (1.2)	< 0.001	< 0.001	< 0.001	ns	< 0.01	ns
14. I feel able to ask my supervisors any question I wish.	4.2 (1.0)	4.1 (0.8)	4.5 (1.1)	4.0 (1.3)	4.1 (1.1)	< 0.01	ns	ns	ns	< 0.05	ns
15. I get the opportunity to provide a rationale for my actions during supervision sessions.	3.7 (1.1)	3.3 (1.0)	4.3 (1.0)	4.3 (0.7)	3.9 (1.1)	< 0.001	< 0.001	< 0.001	ns	< 0.05	< 0.05
16. My problem-solving skills are developing well in this placement.	3.7 (1.0)	3.4 (1.0)	4.3 (0.8)	4.0 (1.0)	3.8 (1.0)	< 0.001	< 0.05	< 0.05	ns	< 0.05	ns
17. I have the opportunity to put my theoretical knowledge into practice in this placement.	3.9 (0.1)	3.7 (1.0)	4.3 (0.8)	4.3 (0.8)	3.9 (0.9)	< 0.001	< 0.001	ns	ns	< 0.05	< 0.05
18. I have the opportunity to learn together with other students in this placement.	3.4 (1.2)	3.3 (1.1)	4.0 (0.9)	3.4 (1.4)	3.1 (1.3)	< 0.001	ns	ns	ns	< 0.001	ns
26. I feel I have influence over my learning in this placement.	3.5 (1.1)	3.2 (1.0)	4.0 (1.0)	4.0 (1.0)	3.6 (1.2)	< 0.001	< 0.001	< 0.05	ns	ns	ns
**Subscale A2: Preparedness for student entry**	21.7 (5.6)	19.2 (4.4)	26.5 (3.5)	25.1 (4.8)	21.6 (6.1)	< 0.001	< 0.001	< 0.001	ns	< 0.001	< 0.01
1. I received useful induction to this placement.	3.7 (1.0)	3.5 (0.9)	4.2 (0.8)	4.1 (0.8)	3.6 (1.1)	< 0.001	< 0.01	ns	ns	< 0.001	ns
2. My supervisors were expecting me when I arrived.	3.8 (1.1)	3.3 (1.0)	4.7 (0.5)	4.5 (0.8)	3.8 (4.0)	< 0.001	< 0.001	< 0.001	ns	< 0.001	< 0.001
9. I have a supervisor to whom I know I can turn.	3.9 (1.2)	3.6 (1.1)	4.5 (0.9)	4.2 (1.1)	3.9 (1.3)	< 0.001	< 0.001	< 0.05	ns	< 0.001	ns
10. I have sufficient access to supervision.	3.8 (1.1)	3.5 (1.0)	4.4 (0.9)	4.1 (1.1)	3.7 (1.3)	< 0.001	< 0.01	ns	ns	< 0.01	ns
11.The supervisors are well prepared for supervising.	3.4 (1.2)	3.0 (1.0)	4.3 (1.0)	4.1 (1.2)	3.6 (1.2)	< 0.001	< 0.001	< 0.001	ns	< 0.001	< 0.05
12. It is clear that my supervisors are familiar with the learning objectives.	3.1 (1.3)	2.3 (1.0)	4.4 (0.8)	4.1 (0.9)	3.2 (1.4)	< 0.001	< 0.001	< 0.001	ns	< 0.001	< 0.01
**Overarching dimension Social participation** (B1 + B2)	35.5 (6.2)	33.6 (5.3)	38.8 (5.3)	38.4 (5.6)	35.3 (7.0)	< 0.001	< 0.001	< 0.05	ns	< 0.01	< 0.05
**Subscale B1: Workplace interaction patterns and student inclusion**	23.0 (4.3)	21.7 (3.8)	25.5 (3.7)	25.1 (4.0)	23.0 (4.7)	< 0.001	< 0.001	< 0.05	ns	< 0.01	< 0.05
7. I have adequate access to computers.	3.6 (1.2)	3.4 (1.1)	4.1 (1.0)	3.7 (1.4)	3.8 (1.1)	< 0.001	ns	< 0.05	ns	ns	ns
8. There is sufficient physical space for the number of students on placement here.	3.8 (1.1)	3.6 (1.1)	4.3 (0.8)	4.0 (1.1)	3.7 (1.1)	< 0.001	ns	ns	ns	< 0.01	ns
19. As a student, I am received in a positive way by the staff here.	4.1 (0.8)	3.9 (0.7)	4.4 (0.8)	4.5 (0.6)	4.0 (0.9)	< 0.001	< 0.001	ns	ns	< 0.05	< 0.01
20. I feel included in the team of people who work here.	3.7 (1.0)	3.5 (1.0)	4.2 (0.9)	4.2 (0.9)	3.8 (1.1)	< 0.001	< 0.001	< 0.05	ns	< 0.05	ns
21. I feel welcome in the staff room/lunchroom here.	4.0 (1.0)	3.7 (0.9)	4.2 (0.9)	4.4 (1.0)	4.0 (1.0)	< 0.01	< 0.001	ns	ns	ns	ns
22.Communication between those working here is good.	3.8 (1.0)	3.6 (0.7)	4.3 (1.1)	4.3 (0.8)	3.7 (1.1)	< 0.001	< 0.001	ns	ns	< 0.01	< 0.05
**Subscale B2: Equal treatment**	12.5 (2.5)	12.0 (2.3)	13.4 (5.3)	13.3 (2.0)	12.3 (2.8)	< 0.001	< 0.01	ns	ns	< 0.05	ns
23. Everyone is treated equally here, regardless of cultural background.	4.3 (0.9)	4.2 (0.9)	4.6 (0.7)	4.7 (0.6)	4.1 (1.1)	< 0.01	< 0.01	ns	ns	< 0.05	< 0.05
24. Everyone is treated equally here, regardless of gender.	4.3 (0.9)	4.1 (0.9)	4.6 (0.7)	4.4 (0.8)	4.3 (0.9)	< 0.01	ns	ns	ns	ns	ns
25. Everyone is treated with the same respect and dignity, regardless of professional background.	3.9 (1.0)	3.7 (1.0)	4.2 (0.1)	4.2 (0.9)	3.9 (1.1)	< 0.01	< 0.01	ns	ns	ns	ns

*Abbreviations*: *UCEEM* Undergraduate Clinical Education Environment Measure, *SD* Standard deviation, *PT* Physiotherapy students, *SLP* Speech-language pathology students, *M* Medical students, *N* Nursing students

^a^The UCEEM total score ranges from 26 to 130. Scale A ranges from 17 to 85; subscale A1 ranges from 11 to 55, and subscale A2 ranges from 6 to 30. Scale B ranges from 9 to 45; subscale B1 ranges from 6 to 30, and subscale B2 ranges from 3 to 15. The p-values show the results of comparisons between the programs, which were calculated using Kruskal–Wallis tests with Dunn–Šidák post-hoc tests

### Overarching dimension experiential learning

The mean score for the overarching dimension experiential learning, based on the subscales of opportunities to learn in and through work and quality of supervision and preparedness for student entry, was 62.8 (SD 13.6; range 59–85) (Table 2). The medical students provided significantly lower ratings for this dimension than the physiotherapy (*p* <  0.001), SLP (*p* <  0.001), and nursing students (*p* <  0.01) did.

#### Subscale and item scores

The medical students rated six items significantly lower (*p* <  0.001) than the students from the other programs did. Three of the items belong to the subscale opportunities to learn in and through work and quality of supervision: *I am encouraged to participate actively in the work here, I receive useful feedback from my supervisors*, and *I get the opportunity to provide a rationale for my actions during supervision sessions*. Three of the items belong to the subscale preparedness for student entry: *My supervisors were* e*xpecting me when I arrived*, *The supervisors are well prepared for supervising*, and *It was clear that my supervisors were familiar with the learning objectives.*

#### Comparisons between semesters

There were few statistically significant differences between the semesters within each program (Table 3). The mean scores for the overarching dimension of experiential learning and for its subscales did not differ between the semesters. The only exception was the subscale of opportunities to learn in and through work and quality of supervision, which the medical students in the 6th semester rated higher than the students in the 10th semester. At the item level, the 6th and 10th semester medical students yielded the greatest difference for *Opportunity to put theoretical knowledge into practice* (*p* <  0.01), in which semester 6 students rated higher. Likewise, the items *My problem-solving skills developed well* and *I felt I had influence over my learning in this placement* were given higher ratings by medical students from the earlier semester (*p* <  0.05). The 6th semester SLP students rated the item *I had the opportunity to learn together with other students* significantly higher than the students from 4th semester did. The 3rd semester physiotherapy students gave higher scores for the item *It is clear that my supervisors were familiar with the learning objectives* (*p* <  0.05), which belongs to the subscale of preparedness for student entry, than the students in semester 6 did.

**Table 3:** Comparisons between semesters for UCEEM scores

UCEEM items	Medical students Mean (SD) S6/S10	Physiotherapy students Mean (SD) S3/S6	SLP students Mean (SD) S4/S6	Nursing students Mean (SD) S3/S6
**Total score**^a^	91.5(12.8)/89.3(17.4)	112.4(15.6)/112.2(9.9)	107.3(14.0)/110.0(20.0)	99.4(20.1)/95.0(21.3)
**Overarching dimension Experiential learning** (A1 + A2)	58.1(9.4)/55.2(12.5)	73.7(11.9)/73.2(7.7)	68.7(11.3)/71.6(14.2)	63.9(14.5)/60.2(14.9)
**Subscale A1: Opportunities to learn in and through work and quality of supervision**	39.0(6.0)/36.0(8.4)*	46.8(8.7)/47.1(4.9)	43.4(7.3)/46.2(9.4)	42.1(9.3)/39.7(9.1)
3. My (work) tasks are relevant to the learning objectives.	3.7(0.8)/3.5(1.0)	4.3(0.8)/4.5(0.6)	4.3(0.8)/4.2(0.8)	4.0(1.0)/4.0(1.1)
4. I am sufficiently occupied with meaningful (work) tasks.	3.3(0.9)/3.0(1.0)	4.0(1.2)/4.2(0.7)	3.7(1.2)/4.3(0.8)	3.5(1.1)/3.5(1.2)
5. My tasks are suitably challenging for my level of knowledge and skills.	3.4(0.9)/3.3(1.1)	4.0(1.3)/ 4.1(0.8)	4.2(0.6)/4.2(1.2)	3.7(1.0)/3.5(1.1)
6. I am encouraged to participate actively in the work here.	3.5(0.7)/3.3(0.9)	4.5(1.0)/4.6(0.6)	4.3(0.9)/4.6(0.6)	3.9(1.1)/3.8(1.0)
13. I receive useful feedback from my supervisors.	3.2(1.0)/2.8(1.1)	4.5(0.7)/4.4(0.8)	3.9(1.0)/3.9(1.0)	3.9(1.2)/3.5(1.2)
14. I feel able to ask my supervisors any question I wish.	4.2(0.7)/4.0(1.1)	4.5(1.2)/4.4(0.9)	4.2(1.1)/4.0(1.4)	4.2(1.1)/3.9(1.2)
15. I get the opportunity to provide a rationale for my actions during supervision sessions.	3.2(0.9)/3.3(1.0)	4.5(1.1)/4.1(0.8)	4.3(0.8)/4.4(0.7)	4.0(1.0)/3.6(1.3)
16. My problem-solving skills are developing well in this placement.	3.6(0.9)/3.2(1.0)*	4.3(0.9)/4.3(0.7)	3.7(1.0)/4.3(1.0)	3.8(1.0) 3.8(1.0)
17. I have the opportunity to put my theoretical knowledge into practice in this placement.	3.9(0.7)/3.4(1.0)**	4.3(0.9)/4.4(0.6)	4.3(0.8)/4.4(0.9)	3.9(0.9) 3.8(0.9)
18. I have the opportunity to learn together with other students in this placement.	3.4(1.1)/3.3(1.2)	4.2(1.0)/3.9(0.9)	2.9(1.2)/3.8(1.4)*	3.2(1.5)/3.0(1.1)
26. I feel I have influence over my learning in this placement.	3.4(0.9)/3.0(1.2)*	3.9(1.1)/4.0(0.9)	3.9(0.9)/4.0(1.1)	3.7(1.2)/3.4(1.2)
**Subscale A2: Preparedness for student entry**	19.1(4.2)/19.1(4.6)	26.9(3.8)/26.1(3.3)	25.7(4.1)/25.3(5.2)	22.0(5.9)/20.8(6.6)
1. I received useful induction to this placement.	3.5(1.0)/3.4(0.9)	4.0(1.0)/4.4(0.5)	3.8(0.8)/4.3(0.8)	3.8(1.1)/3.4(1.2)
2. My supervisors were expecting me when I arrived.	3.2(1.0)/3.3(0.9)	4.7(0.6)/4.7(0.5)	4.7(0.9)/4.6(0.6)	3.7(1.3)/3.9(1.2)
9. I have a supervisor to whom I know I can turn.	3.6(0.8)/3.6(1.1)	4.7(0.9)/4.4(0.9)	4.6(0.5)/4.1(1.3)	3.9(1.2)/3.7(1.4)
10. I have sufficient access to supervision.	3.5(0.9)/3.5(1.2)	4.5(0.9)/4.2(0.8)	4.3(1.2)/4.1(1.1)	3.8(1.2)/3.5(1.2)
11.The supervisors are well prepared for supervising.	3.0(0.9)/2.9(1.1)	4.4(1.1)/4.2(0.8)	4.3(1.0)/4.0(1.3)	3.6(1.2)/3.4(1.2)
12. It is clear that my supervisors are familiar with the learning objectives.	2.3(0.9)/2.4(1.1)	4.6(0.8)/4.2(0.9)*	4.0(0.8)/4.2(0.9)	3.3(1.3)/3.0(1.4)
**Overarching dimension Social participation** (B1 + B2)	33.3(4.6)/33.9(6.2)	38.7(5.8)/39.0(5.0)	38.6(4.6)/38.4(6.3)	35.8(6.5)/34.4(7.9)
**Subscale B1: Workplace interaction patterns and student inclusion**	21.5(3.3)/21.7(4.4)	25.6(3.8)/25.4(3.6)	24.9(3.3)/25.4(4.6)	23.4(4.5)/22.3(5.1)
7. I have access to computers.	3.4(1.1)/3.3(1.1)	4.4(0.8)/3.8(1.0)*	3.3(1.2)/4.1(1.5)	3.9(1.0)/3.7(1.2)
8. There is sufficient physical space for the number of students on placement here.	3.7(1.0)/3.5(1.2)	4.6(0.6)/4.1(1.0)	4.3(0.9)/3.9(1.1)	3.7(1.2)/3.7(1.1)
19. As a student, I am received in a positive way by the staff here.	3.9(0.6)/3.8(0.9)	4.3(0.8)/4.5(0.7)	4.5(0.7)/4.6(0.6)	4.1(0.9)/3.9(1.1)
20. I feel included in the team of people who work here.	3.4 (0.9) / 3.6 (1.1)	4.0 (1.0) / 4.4 (0.8)	4.3 (0.6) / 4.1 (1.0)	3.8 (1.0) / 3.7 (1.2)
21. I feel welcome in the staff room/lunchroom here.	3.7(0.8)/3.8(1.0)	4.0(1.2)/4.3(0.9)	4.3(0.7)/4.3(1.2)	4.1(1.0)/3.8(1.0)
22. Communication between those working here is good.	3.5(0.8)/3.7(0.6)*	4.3(0.7)/4.3(0.7)	4.2(0.9)/4.3(0.8)	3.9(0.9)/3.3(1.4)*
**Subscale B2: Equal treatment**	11.7(2.2)/12.2(2.4)	13.1(2.6)/13.6(1.8)	13.7(1.9)/13.1(2.2)	12.5(2.6)/11.9(3.1)
23. Everyone is treated equally here, regardless of cultural background.	4.1(0.8)/4.2(1.1)	4.5(0.8)/4.6(0.6)	4.7(0.5)/4.6(0.7)	4.2(1.9)/4.1(1.2)
24. Everyone is treated equally here, regardless of gender.	4.1(0.8)/4.2(1.0)	4.5(0.8)/4.7(0.5)	4.6(0.7)/4.3(0.9)	4.3(0.8)/ 4.1(1.0)
25. Everyone is treated with the same respect and dignity, regardless of professional background.	3.5(1.0)/3.9(1.0)*	4.2(1.1)/4.2(0.9)	4.4(0.8)/4.2(0.9)	4.1(1.0)/3.7(1.2)

*Abbreviations*: *UCEEM* Uundergraduate Clinical Education Environment Measure, *SD* Standard deviation, *S* Semester, *SLP* Speech-language pathology

^a^The UCEEM total score ranges from 26 to 130. The overarching dimension experiential learning ranges from 17 to 85. Subscale A1 ranges from 11 to 55, and subscale A2 ranges from 6 to 30. The overarching dimension social participation ranges from 9 to 45. Subscale B1 ranges from 6 to 30, and subscale B2 ranges from 3 to 15. The p-values show the results of comparisons between the semesters, which were calculated using the Mann-Whitney U test. * *p* < 0.05; ** *p* < 0.01

### Overarching dimension social participation

The mean score for the overarching dimension social participation based on the subscales of workplace interaction patterns and student inclusion and equal treatment was 35.5 (SD 6.2; range 13–45) (Table 2). Medical students provided significantly lower ratings for the dimension than the physiotherapy (*p* <  0.001), SLP (*p* <  0.001) and nursing (*p* <  0.05) students did*.*

#### Subscale and item scores

Within the subscale of workplace interaction patterns and student inclusion, the medical students rated three items significantly lower (*p* <  0.001) than the physiotherapy and SLP students did: *As a student, I am received in a positive way by the staff here*, *I feel included in the team of people who work here*, *and Communication between those working here is good.* The highest ratings within the subscale of equal treatment were found for the items *Everyone was treated equally regardless of cultural background* and *Everyone was treated equally regardless of gender* (Table 2). The medical students rated equal treatment regardless of cultural or professional background lower (*p* <  0.01) than physiotherapy and SLP students did.

#### Comparisons between semesters

The mean scores for the overarching dimension social participation and its subscales did not differ significantly between the semesters. However, item comparisons showed differences within the subscale of workplace interaction patterns and student inclusion. Third-semester nursing students scored *Communication between those working here is good* significantly higher (*p* <  0.05) than those students in the more advanced semesters did. Conversely, the medical students in 6th semester scored the same item significantly lower (*p* <  0.05) than the students in semester 10 did. The item *Everyone was treated with the same respect and dignity, regardless of professional background* (*p* <  0.05), which belonged to the subscale of equal treatment, was rated lower by the medical students in 6th semester than those in 10th semester.

### Internal consistency

The internal consistency of the four subscales was good. The Cronbach’s alpha was 0.919 for opportunities to learn in and through work and quality of supervision; that for preparedness for student entry and equal treatment, as well as that for workplace interaction patterns and student inclusion, was 0.812.

## Discussion

The aim of this study was to describe and compare students’ perceptions of their CLEs across four undergraduate programs by using the recently developed UCEEM questionnaire.

The total UCEEM scores and the scores for the overarching dimensions were high across the study, indicating that the students’ perceptions of their CLEs were quite positive across different study programs. However, the students from the medical and nursing programs rated their CLEs lower than the students from the physiotherapy and SLP programs did. Importantly, at several subscale and item levels, the medical students provided significantly lower ratings for their CLE than the students from the other programs did. There were few significant differences between the semesters within each program.

This study was designed not only to explore the overall CLE but also to describe the possible differences between the studied programs. An overall positive perception of the CLE was shared by the students from all the programs, which was indicated by the high mean total UCEEM scores. However, one unanticipated finding was the significant differences between the programs. While the ratings between the physiotherapy and SLP students did not differ from each other, they both rated their CLEs significantly higher than the medical and nursing students did. To the best of our knowledge, similar results have not been previously reported. There are a number of plausible explanations for our results. For instance, the differences may be partly due to the smaller number of students per semester in the physiotherapy and SLP programs, resulting in fewer students per placement and supervisor. Another explanation could be the length of the clinical placements. Physiotherapy and SLP students could have placements as short as 1 week but usually longer. Clinical education for medical students conventionally consists of fragmented short-term encounters with mainly acute-care patients with an array of clinical supervisors and, therefore, often lacks continuity [35].

Communities of practice develop around the things that matter to its members and to the organizations that support such communities [36]. With shorter placements, as is the case with the medical students in this study, there is little time to gradually move from peripheral participation, in which they are least connected with the community, to engagement with the core health care team. A model with longitudinal integrated clerkships has been introduced to overcome the shortcomings with frequent changes in disciplines and to increase continuity [37]. Students have rated this model highly [38, 39], and evidence shows that teachers also find the longitudinal relationships between them and students satisfactory, but the cost-effectiveness and long-term outcomes are still under discussion [40]. It is worth noting, however, that the total scores were fairly high across the study, which may indicate particular strengths within certain programs rather than particular weaknesses.

With regard to the individual subscales, the perceptions of the opportunities to learn in and through work and quality of supervision as well as preparedness for student entry, both belonging to the dimension of experiential learning, showed the greatest disparity between the programs. The mean scores for these subscales were more than 6 points higher for physiotherapy and SLP students than for medical students, suggesting that the former perceived items, such *as Encouraged to participate in the work* and *Receiving useful feedback from my supervisors*, more positively than the medical students did. The results of Strand et al. [30] and Roberts et al. [31], who studied medical students in the Swedish and UK settings, respectively, were similar, despite marginal differences in the studied populations. Strand et al. [30] chose a broad sample—a population comparable to ours—to investigate students in semesters 6–10, whereas Roberts et al. [31] included final-year medical students in their analysis. Previous studies have emphasized that the CLE quality often depends on the supervision. In a comprehensive study of 2500 healthcare students who completed clinical placements at a university hospital, Pitkänen et al. [5] showed that supervisory relationships had a significant effect on students’ CLE experiences. Further, these authors found that the relationship was particularly good amongst students who had named supervisor with whom they discussed the learning outcomes. It is challenging for supervisors to encourage students to participate in clincal work and give them useful feedback if the supervisor and the student encounters are short and lack continuity. Unfortunately, the supervision models and the feedback strategies used by the different programs and placements were not investigated in the present study.

At the item level, a disappointing finding was the medical students’ low scores for *Supervisors’ familiarity with the learning objectives*. A well-designed course aligns the activities with the learning outcomes and assessment [41]. Thus, supervisors need to know the learning outcomes in order to help students meet their learning needs and assess whether the tasks are relevant for them. There are only a few studies available for comparison. The DREEM questionnaire [23] includes a similar item, but it focuses on whether the student, not the supervisor, is familiar with the learning objectives. In the study of Roberts et al. [31], in which more than 100 medical students were involved, supervisors’ familiarity with the learning objectives was higher than that in our study, but the authors studied final-year medical students from four specialties. The length of each rotation in their study was 8 weeks, whereas the medical students in our university had rotations as short as a couple of days and no longer than two to 3 weeks. It is likely that the medical students in our study met a larger number of supervisors during their rotations, which might have contributed to knowledge variations regarding the overarching learning objectives. It is also plausible that the supervision structure differs between the settings and varies from rotation to rotation, which has also been confirmed by previous research [42] but not investigated in the current study. Nonetheless, the learning outcomes should determine the content of the curriculum. When supervisors know the learning objectives, they can recognize students’ learning needs and ensure relevant learning experiences [43].

Clinical placements provide students with the opportunity to socialize into their future profession and learn interprofessional clinical practice. Our results for the overarching dimension social participation comprising the subscale of workplace interaction patterns and student inclusion indicate that the students were received in a positive way and welcomed to the staff room. These results reflect those of Strand et al. [30] and Roberts et al. [31] who also found comparable scores for these subscales. An important finding in the other subscale level here was the relatively high scores for equal treatment. This refers to perceived discrimination in the workplace, in general [30], and may involve discrimination or harassment based on race, religion, ethnicity, or gender or include aspects of mistreatment [44]. Importantly, perceptions of a discriminatory climate negatively affect educational outcomes [45]. We did not find substantial differences between male and female students, which is encouraging. These results are consistent with those of Strand et al. [30] and Roberts et al. [31], but contradictory findings in a comprehensive study among nursing and medical students from New Zeeland show that bullying and harassment of students in health professional education are widespread problems [46]. However, the prevalence of bullying and harassment seems to vary, ranging, for instance, from 25% among physiotherapy students [47] to 91% among medical students [48] and 90% among nursing students [49]. This inconsistency may be due to several factors, such as the different definitions of bullying and harassment and the use of different instruments [46]. To our knowledge, with the exception of the UCEEM, few instruments measure equal treatment aspects in the CLE. The Postgraduate Hospital Educational Environment Measure [24] measures comparable aspects, whereas the Dutch Residency Educational Climate Test [27] and the Scan of Postgraduate Educational Environment Domains [28] measure respectful attitudes. Nevertheless, all these instruments are created for postgraduate education. It is important to measure equal treatment aspects in future evaluations in order to provide students with a safe and inclusive CLE, and the UCEEM seems to be a suitable tool for this purpose.

Perceptions of the CLE showed certain variations between students from earlier and later semesters, with younger students from the medical and nursing programs rating the items slightly higher compared with the students from the later semesters. However, the variations did not follow a consistent pattern across the programs, so no firm conclusions can be drawn.

There are no studies that have used UCEEM for comparison, but similar inconsistency between semesters has been shown in a comprehensive Australian study that used the DREEM and that included more than 500 students from eight different health science courses [20]. It could be speculated that students from later semesters are more experienced and, therefore, more demanding. It is also plausible that older students show training fatigue [50] and are less enthusiastic. Rotthoff et al. [51] previously assumed that the perception of a deterioration of the LE is not due exclusively to educational delivery but also to individual factors, such as aging, becoming more autonomous, and becoming more critical.

This study is unique in that it covers 280 students from the early and later semesters from four study programs. The diversity of the environments and the significant results for many items are the strengths of the study. However, several limitations warrant discussion. One limitation has to do with the UCEEM questionnaire. It was designed for medical students but was applied to physiotherapy, SLP, and nursing students based on the assumption that the CLE is essentially the same for all undergraduate students. In addition, the questionnaire was relatively new, making comparisons with other studies difficult. There were no recommendations for the analysis or presentation of the results [30]. Second, there is controversy regarding how Likert data should be analyzed, which is a topic that has been debated in the scientific literature for nearly 50 years [52]. From a statistical point of view, utilizing means and parametric statistical testing on ordinal data from Likert data as used in this study may not be acceptable. However, according to Carifio and Perla [52], the discussion on how Likert responses should be analyzed strongly posits, that while Likert items may well be ordinal, Likert scales, consisting of sums across many items, will be interval. It is therefore suitable to summarize the scores produced from Likert responses using means and standard deviations as well as parametric statistical techniques. Third, the UCEEM is constructed employing a 1–5 Likert response format instead of a 0–4 scale, and therefore performing percentage comparisons, as recommended by Dimoliatis et al. [53], was difficult. Lastly, the response rates were satisfactory for three of the programs, albeit quite low for the nursing program, wherefore the total response rate was moderate. Thus, an important limitation of this study concerns the response rate for the nursing students, herefore, the results have to be interpreted with caution. Nevertheless, we regard the sample as being of sufficient variety and size to provide valuable data.

Further research should be undertaken to investigate the conditions of clinical supervisors. A more detailed description of supervisor training and the model of supervision in different programs would be valuable. Such a study could also aim to specify what students identify as central to good supervision and preparation for student entry. A detailed description of how different programs work to make supervisors more attentive to the intended learning outcomes that are central for clinical training should be made. Our results likewise raise the question of the impact of group size variations and the length of clinical placements in different study programs. These aspects were not explored in this study but should be investigated further.

## Conclusion

The students generally hold positive perceptions toward their CLEs, suggesting that the environments across the programs met the educational needs of the students. However, we found several significant differences between the programs in favor of the smaller programs of physiotherapy and SLP. In several aspects, the medical students provided significantly lower ratings of their CLE compared with the other students. The low ratings for supervisors’ familiarity with the learning objectives underscore the need to ensure that the prerequisites for optimal supervision are met.

## Data Availability

The datasets generated and/or analyzed during the current study are available from the corresponding author on reasonable request.

## Abbreviations

CLE: Clinical learning environment
CoP: Communities of Practice
DREEM: Dundee Ready Education Environment Measure
ECTS: European Credit Transfer and Accumulations system
LE: Learning environment
IRR: Item response rate
PT: Physiotherapy
SLP: Speech-language pathology
SPSS: Statistical Package for the Social Sciences
UCEEM: Undergraduate Clinical Education Environment Measure
